# Matrix-Assisted Laser Desorption/Ionisation Mass Spectrometry Imaging in the Study of Gastric Cancer: A Mini Review

**DOI:** 10.3390/ijms18122588

**Published:** 2017-12-01

**Authors:** Andrew Smith, Isabella Piga, Manuel Galli, Martina Stella, Vanna Denti, Marina Del Puppo, Fulvio Magni

**Affiliations:** Department of Medicine and Surgery, University of Milano-Bicocca, Clinical Proteomics and Metabolomics Unit, 20854 Vedano al Lambro, Italy; andrew.smith@unimib.it (A.S.); isabella.piga@unimib.it (I.P.); m.galli27@campus.unimib.it (M.G.); m.stella12@campus.unimib.it (M.S.); v.denti@campus.unimib.it (V.D.); marina.delpuppo@unimib.it (M.D.P.)

**Keywords:** gastric cancer, MALDI imaging, proteomics, metabolomics, lipidomics

## Abstract

Gastric cancer (GC) is one of the leading causes of cancer-related deaths worldwide and the disease outcome commonly depends upon the tumour stage at the time of diagnosis. However, this cancer can often be asymptomatic during the early stages and remain undetected until the later stages of tumour development, having a significant impact on patient prognosis. However, our comprehension of the mechanisms underlying the development of gastric malignancies is still lacking. For these reasons, the search for new diagnostic and prognostic markers for gastric cancer is an ongoing pursuit. Modern mass spectrometry imaging (MSI) techniques, in particular matrix-assisted laser desorption/ionisation (MALDI), have emerged as a plausible tool in clinical pathology as a whole. More specifically, MALDI-MSI is being increasingly employed in the study of gastric cancer and has already elucidated some important disease checkpoints that may help us to better understand the molecular mechanisms underpinning this aggressive cancer. Here we report the state of the art of MALDI-MSI approaches, ranging from sample preparation to statistical analysis, and provide a complete review of the key findings that have been reported in the literature thus far.

## 1. Introduction

Gastric cancer (GC) develops from the lining of the stomach and is the fifth most common malignancy worldwide [1]. The prognosis of patients with this cancer is related to tumour extent; often early stages of the disease can be asymptomatic, and consequently late diagnosis in advanced stages makes treatment less likely to succeed and reduces patients’ chances of survival. The general prognosis is in fact rather grim for gastric cancer patients, with less than 25% of patients surviving at the five-year time-point following diagnosis [2]. Given the high morbidity and mortality rate, the study of GC represents a pressing area of clinical research and much work is ongoing. However, our comprehension of the mechanisms underlying the development of gastric malignancies is still lacking. Given the breadth of modern analytical instrumentation now at our disposal, the detection of early diagnostic biomarkers of GC may not be a distant hope and such findings could be used to elucidate potential pathways for tailored therapeutic treatment.

Mass spectrometry (MS)-based techniques have become some of the most prevalently employed analytical strategies for the detection and identification of endogenous biomolecules in tissue. The application of these techniques is now commonplace in clinical research [3,4,5] and the mass spectrometric detection of pathologically significant molecules has already shown promise in the study of gastric cancer, providing greater insights into the molecular aspects of the disease and aiding in the identification of candidate biomarkers [6]. Furthermore, the emergence of modern mass spectrometry imaging (MSI) techniques has further revolutionised this area of research. Using MSI, the chemical specificity of MS can be combined with the imaging capabilities offered by optical microscopy in order to simultaneously detect the distribution of hundreds, if not thousands, of biomolecules directly in situ, making it an ideal discovery method for new potential biomarkers for gastric cancer.

Matrix-assisted laser desorption/ionisation (MALDI) remains the most widely applied MSI technique owing to its capability to analyse a wide range of analyte classes (xenobiotics, metabolites, lipids, and proteins) [5]. In particular, the MS-imaging of proteins has been readily performed given their significant role in a large number of pathways involved in defective cellular signalling cascades. Therefore, the ability to spatially resolve the localisation of a number of proteins within the same section of pathological tissue can enable the detection of pathological processes, and, ultimately, define biomarker candidates. Additionally, it has also become increasingly common for the distribution of lipids and metabolites to be recorded by MALDI-MSI, owing in particular to their ease of analysis. Furthermore, there is an ever-increasing body of evidence to suggest that these small molecules play a significant role in biological systems and, as such, are heavily involved in disease pathogenesis. Finally, MALDI-MSI is now readily used to monitor the distribution of xenobiotics and their metabolites within tissue, establishing itself as an invaluable tool in drug distribution studies [7].

In this review, we provide a concise overview of the methodological aspects of MALDI-MSI and summarise how the technique has been used to advance gastric cancer research for the purpose of biomarker detection and monitoring treatment response.

## 2. Matrix-Assisted Laser Desorption/Ionisation-Mass Spectrometry Imaging (MALDI-MSI) in a Nutshell

MALDI-MSI applied to thin mammalian tissue sections was formally introduced in 1997 and its use has increased exponentially in recent years [8]. The technique relies on the use of a MALDI matrix, which consists of small organic molecules that are designed to absorb the energy of a pulsed laser beam. These molecules commonly possess a suitable chromophore, usually in the form of an aromatic core, and it is this property of the matrix that facilitates the absorption of the UV laser energy. When this matrix is applied to the surface of a sample, it promotes the formation of a ubiquitous layer of co-crystals, which incorporates both matrix and analyte molecules in its network. When the laser beam is applied to the surface of the sample, the absorbed energy leads to rapid desorption of both the matrix and analyte crystals and subsequent “soft” ionisation [3].

Typical MALDI-MSI analysis is most commonly performed on tissue sections that have been sectioned and mounted onto electrically conductive glass slides, such as those coated with indium tin oxide (ITO) [9]. For protein, lipid, xenobiotics and metabolite imaging, the analysis is most commonly performed using fresh-frozen (FF) tissue [10,11].

Regarding the imaging of drugs and products of drug metabolism, MALDI-MSI has been readily used within the pharmaceutical community for the purpose of drug discovery and development [12]. The monitoring of the spatial distribution of drugs and their metabolites in order to evaluate a drug’s absorption properties, as well as the characterisation of a drug’s delivery and penetration in a target organ, represent some examples of how MALDI-MSI tools have been successfully applied in this field [13,14]. In addition to qualitative MALDI-MSI approaches, the ability to obtain absolute quantitative information by MALDI-MSI for drug analysis, by applying internal standards, has recently been further investigated [15,16].

**Figure 1:**
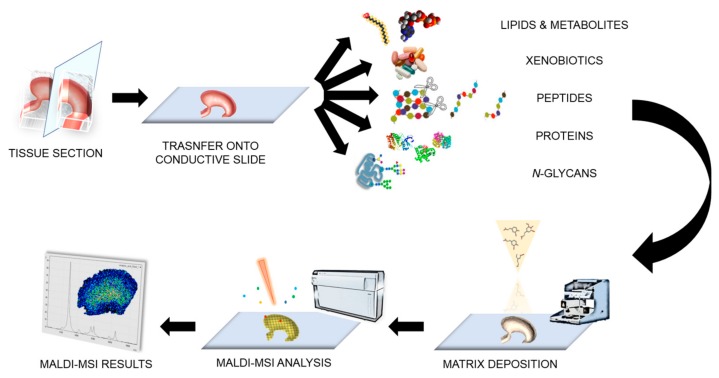
Illustration of the workflow for the matrix-assisted laser desorption/ionisation-mass spectrometry imaging (MALDI-MSI) analysis.

In the case of protein imaging, formalin-fixed paraffin-embedded (FFPE) tissue is now also readily employed [17]. FFPE tissue accounts for a large percentage of the patient samples collected and stored in medical centres [18] and thus represents a potential gold mine of information for histopathological studies involving MALDI-MSI. It also facilitates multi-centric studies using tissue specimens from numerous tissue banks [19,20]. However, the sample preparation for protein imaging of FFPE tissue is more complex and requires an antigen retrieval step followed by tryptic digestion prior to MALDI-MSI analysis. Metabolite imaging has also been conducted on FFPE tissue [21]; however, it has been less extensively investigated with respect to proteins. Finally, a number of groups have focused on the analysis of *N*-glycans in tissue [22,23], demonstrating that it is possible to monitor the distribution of both *N*-glycans and proteins within the same tissue section [23]. The potential to monitor *N*-glycans, one of the most common post-translational modifications, may significantly advance MALDI-MSI investigations in gastric cancer given their fundamental role in many cellular processes and their establishment as clinical biomarkers [23]. A general overview of the MALDI-MSI sample preparation and analysis workflow is given in Figure 1.

### 2.1. Sample Preparation

Particular attention to detail must be paid during the collection of FF tissue, as negligence during the sample collection can lead to degradation and delocalisation of the analytes of interest. The method most commonly employed during collection is snap-freezing using liquid nitrogen; however, this procedure can damage tissue morphology if it cools at different rates. This can be overcome to some degree by lightly wrapping the tissue in aluminium in order for it to cool at a more uniform rate [24]. Alternatively, Goodwin et al. recommend the use of ethanol or isopropanol solutions at temperatures of ≤−70 °C [25]. Once snap-frozen, FF tissue sections can be maintained at −80 °C for up to a year without evidence of degradation [3,24,26]. Prior to matrix application, tissue washes are also performed in order to remove any molecules that may interfere with the ionisation of the target analytes, including any compounds used during the sectioning procedure. Standard protocols for protein MS-imaging recommend washing the tissue sequentially in increasing concentrations of ethanol, whilst, for example in tissue with a high lipidomic content, washing this tissue with chloroform or xylene can improve protein detection [24,25,27]. Conversely, different washing protocols should be used if the intended analytes are not proteins, e.g., ethanol (70%) with the addition of ammonium acetate (NH_4_Ac) is recommended for the desalting of tissue prior to lipidomic analysis [28].

Regarding FFPE tissue, metabolite MS-imaging requires tissue immersion in xylene, or a similar organic solvent, in order to remove any paraffin [21]. Protein MS-imaging, however, requires a more complex procedure [4,29,30]. Following paraffin removal, tissue rehydration is then performed prior to antigen retrieval. The antigen retrieval step is generally performed at 97 °C whilst immersed in a buffer solution that most commonly contains either Tris-HCl or citric acid [31,32,33], and is required in order to break the methylene bridges that have formed between amino acids during the fixation process. Whilst enzymatic digestion is conventionally performed in solution for proteomic investigations, here the spatial integrity of the proteins is required, and thus the procedure is performed in situ. However, the hydrophobic nature of certain proteins renders them proteolytically resistant to digestion and ultimately limits the peptide yield when performed in this manner. The addition of detergents to the trypsin solution can improve solubilisation by unfolding the proteins, increasing the number of possible enzymatic cleavage sites. A number of detergents have been shown to be compatible with MALDI-MSI analysis, such as *N*-Octanoyl-*N*-methylglucamin (MEGA-8) and RapiGest SF (Waters Corporation, Manchester, UK) [34,35], and significantly improved peptide yield as well as signal intensity, facilitating a greater number of peptide identifications whilst using a bottom-up approach. Alternatively, enzymatic digestion can be performed using *N*-glycosidase F (PNGase F) in order to visualise the distribution of *N*-glycans that are associated with different pathological states of tissue [23].

Matrix deposition plays a crucial role in MALDI-MSI experiments and can limit the true spatial resolution that can be achieved. The general aim of the co-crystallisation process is to maximise analyte extraction whilst at the same time limiting the degree of lateral diffusion, which is equally important to the choice of matrix [9]. Wet matrix deposition methods, involving the use of automated spotters [36] and, in particular, sprayers [37], are particularly efficient for the extraction of proteins and peptides and commonly lead to the formation of crystals of between 10 and 50 μm in diameter. On the other hand, solvent-free matrix deposition involving sublimation has surged in popularity for the analysis of lipids and metabolites due to its ability to deposit a uniform coating of fine matrix crystals that are only a few microns in diameter [38]. Therefore, sublimation represents a highly cost-effective approach to matrix deposition that is both reproducible and compatible with high spatial resolution MALDI-MSI [9]. In contrast to sublimation methods that deposit dry matrix onto the surface of the tissue section, microscope glass slides can also be pre-coated with a MALDI matrix prior to tissue mounting [39]. This has also been shown to be a high-throughput approach that can be effective for the analysis of both proteins [39] and low molecular weight compounds, such as lipids and metabolites [40].

Depending on the target analyte of choice, a number of different matrices can be used. For example, DHB (2,5-Dihydroxybenzoic acid), sinapinic acid (SA; 3,5-dimethoxy-4-hydroxycinnamic acid) and α-CHCA (α-cyano-4-hydroxycinnamic acid) are the most common matrices of choice for the extraction of low molecular weight proteins, peptides, and lipids (1–20 kDa) [41]. However, the addition of hexafluoroisopropanol (1,1,1,3,3,3-hexaluoro-2-propanol) and 2,2,2-trifluoroethanol to the matrix solution [42], along with the use of detectors designed for the detection of higher molecular weight analytes, has been shown to enhance the potential to detect higher molecular weight proteins whilst using SA (up to 110 kDa) [43]. Alternatively, ferulic acid (3-(4-hydroxy-3-methoxy-phenyl)-prop-2-enoic acid) may also be used for the extraction of high molecular weight proteins (up to 140 kDa) [44]. Additionally, ionic matrices such as CHCA/aniline (CHCA/ANI) and CHCA/*N*,*N*-dimethylaniline (CHCA/DANI) have been employed to obtain a more ubiquitous matrix layer and enhance the detection of protein signals [45]. For metabolite imaging, 9-aminoacridine (9AA) is often employed and the mass spectrometer is set in negative-ion mode [46]. In view of the rapid evolution in mass spectrometric instrumentation, the search for novel matrices and matrix deposition protocols has also come to the fore. For example, Garate et al. demonstrated the use of MBT (2-mercaptobenzothiazole) and DAN (2,5-diaminonapthalene) as MALDI matrices that produced very small crystals and were not a limiting factor during the acquisition of MALDI-MS images with pixel sizes as low as 5 μm [47].

### 2.2. Instrumental Advancements

MALDI mass spectrometry instrumentation has rapidly evolved in recent years, offering ever more mass resolution and increased sensitivity. In fact, state-of-the-art MALDI-MS instrumentation enables the generation of individual spectra with intensities measured at 25,000–50,000 *m*/*z*-bins for ToF MS and even greater than 1,000,000 *m*/*z*-bins for Fourier-transform ion cyclotron resonance (FTICR) MS measurements [48]. These advancements have enabled more comprehensive analysis and the better resolution of species with similar *m*/*z* values. In fact, modern MALDI-FTICR-MS instrumentation, as well as MALDI linear ion trap (Orbitrap), can enable the unequivocal identification of certain analytes (particularly for small molecular weight compounds such as lipids, drugs and metabolites) based on their accurate mass alone [49,50]. Furthermore, the addition of a separate dimension, the drift time, to quadrupole-ToF and ion mobility instrumentation can overcome the inability of MALDI-ToF instruments to differentiate isobaric ions, enabling the detection of a higher number of peaks [51]. Notwithstanding this rapid evolution, several technical issues related to MALDI-MSI still need to be improved, such as spatial resolution and sensitivity. However, next-generation instruments are beginning to address these limiting factors [52], not only improving spatial resolution and sensitivity, but also increasing the spectral acquisition rate as well as minimising pixel-to-pixel variability, facilitating higher quality and more robust analysis. Continuing in this vein, MALDI-MSI will be able to not only analyse single cells, but also potentially delve deeper and analyse at a subcellular level, enabling the intra-cellular proteome to be investigated. Furthermore, it will also be possible to routinely generate three-dimensional MALDI-MS images in order to obtain a snapshot of the pathological state of an entire organ by combining MALDI-MS images of consecutive tissue sections and reconstructing a three-dimensional representation using the appropriate (and currently available) software [53,54,55].

### 2.3. Statistical Analysis and Data Elaboration

**Figure 2:**
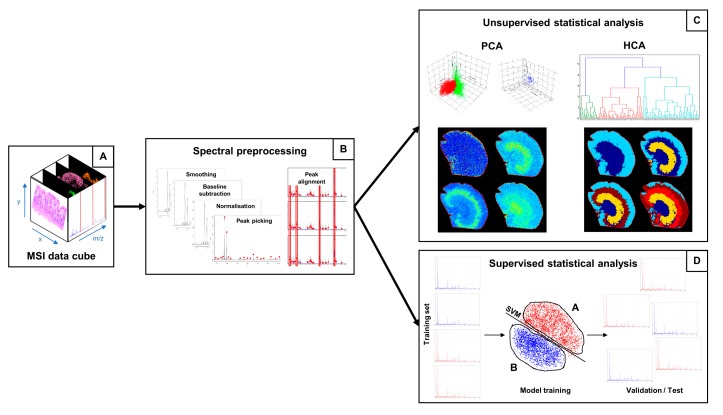
A schematic overview of the MSI data elaboration workflow. (**A**) Data cube; (**B**) the series of spectra pre-processing steps; (**C**) unsupervised and (**D**) supervised statistical analysis performed on a spectra dataset. MSI, mass spectrometry imaging; PCA, principal component analysis; HCA, hierarchical clustering analysis; SVM, support vector machine.

MALDI-MSI records the presence and relative abundance of a great variety of molecules on tissue, allowing the localisation and spatial distribution of such molecules to be visualised. For each pixel of the digitalised tissue image, a mass spectrum is acquired, generating a so-called “data cube” (Figure 2A), a tensor in which the two spatial dimensions (*x* and *y* axes) of the digitalised tissue section are combined with a third dimension consisting of the mass-to-charge ratio (*m*/*z*) of the molecules present within the tissue section. Depending on the spatial resolution and the number of data points (sampling rate) per spectrum, a MALDI-MSI dataset can be of several gigabytes, even terabytes. Therefore, efficient statistical methods for data mining must be employed in order to extract information from the spectral data [56].

Before proceeding with the statistical analysis, however, a series of pre-processing steps are required in order to remove the analytical variability connected with the chemical impurities present in the samples and the electronic nature of the mass spectrometric instrumentation [57,58]. These steps adequately prepare the MS data for statistical analysis and enhance the biological information present within the data (Figure 2B) [59]. Smoothing, performed by employing algorithms such as the Savitzky–Golay filter and the moving average window, aims at discarding the fluctuations in the spectrum mainly due to the electronic nature of the mass spectrometer: this process enhances and eases the peak detection phase, since false positive peaks corresponding to electrical noise are discarded. Baseline subtraction, performed by algorithms such as TopHat, iterative convolution and convex hull, ensures that the spectra all lie on the *x*-axis and all the peak intensities are estimated from the *x*-axis itself. Normalisation multiplies the intensity of the data points of the spectra by a scaling factor in order to bring the intensity scale (merely related with the analogue-digital conversion of the signal) within the same range and therefore make analyses more reproducible [60]: the total ion count (TIC) method divides the spectrum intensities by the sum of all the intensity values for that spectrum; the root mean square (RMS) method divides the spectrum intensities by the square root of the sum of the intensity values for that spectrum squared; the median method divides the spectrum intensities by the median intensity of that spectrum. Finally, peak picking extracts the information regarding the peaks present within the mass spectrum, in the form of *m*/*z* and intensity pair values. After peak maxima have been aligned to each other in order to account for fluctuations in the peak values among the spectra of the dataset related with the peak picking process, the data can be submitted to statistical analysis. Mostly, machine learning algorithms are employed for statistical analysis of the data cube, and, depending on the data provided and on the aim of the data mining process, unsupervised or supervised approaches are carried out (Figure 2C,D) [61].

Unsupervised learning takes unlabelled data as input, i.e., data in which the outcome is not known; by the exploitation of the intrinsic information present in the data, clustering operations are performed in order to highlight hidden structures and/or patterns within the data and are achieved by estimating the similarities among data observations [62]. However, these approaches can also be used in a partially supervised manner, in such a way that the outcome of each observation is preserved during the unsupervised analysis but not taken into account by the algorithm, which performs its operations blind.

Examples of the unsupervised methods for statistical analysis that have been applied in the case of MALDI-MSI gastric cancer datasets are hierarchical clustering analysis (HCA), principal component analysis (PCA) and t-distributed stochastic neighbour embedding (t-SNE). Hierarchical clustering analysis (HCA) estimates the pairwise distance among data observations and generates a dendrogram, in which the observations are grouped under the same nodes based on their similarity to each other [62]. In mass spectrometry imaging, data observations correspond to individual spectra and pixels are associated with spectra; therefore, pixels corresponding to spectra under the same node can be coloured in the same way, generating an unsupervised segmentation tissue image, which can highlight areas of interest on a molecular basis without a priori knowledge regarding the presence of such areas in the tissue section [63]. Therefore, the MSI approach has the potential to aid the diagnostic process by bringing areas of tissue to the attention of the pathologist and highlighting the molecular alterations, even if they do not correlate with cyto-morphological features. Principal component analysis (PCA) is a mathematical technique that aims at reducing data dimensionality whilst preserving the information present within the data [64]. PCA provides an overview of the entire spectral dataset by generating new variables (called principal components, PC) from the linear combination of the spectral features (i.e., peaks): since the PCs are generated orthogonally to one another, no redundancy among the new variables is present and PCs are sorted according to the amount of variance that is retained from the original dataset. This is done in such a way that an overview of almost all the information present within the data can be obtained by looking at the first principal components. The output of a PCA consists of a score chart and a loadings plot: the former places data observations in a 2D or 3D graph according to the score of the PCs, allowing the degree of similarity among the spectra to be evaluated according to their distribution/clustering in the chart; the latter, by resembling the distribution of the former, allows us to evaluate which feature contributes more significantly in driving the distribution/clustering of data observations in the score chart. By combining the two plots, not only is it possible to determine whether the data is capable of discriminating among different classes, but also putative signals of interest can be highlighted for further investigation. t-SNE is a non-linear dimensionality reduction technique that aims at reducing the number of dimensions to two or three in such a way that a 2D or 3D visualisation is easily computed [65]: each n-dimensional data point is mapped to a two- or three-dimensional point in such a way that similar observations correspond to close points in the mapped space. While PCA generates new variables by computing a linear combination of features, t-SNE retains all the features as they are in order to perform the computations. In the case of spectral datasets, t-SNE can be applied by employing either all the individual peaks or only the spectral data points.

On the other hand, supervised learning aims at employing algorithms, referred to as classifiers, which learn from labelled data, i.e., data in which the outcome is known, in order to exploit known features (which correspond to peaks in the mass spectrometry imaging dataset) to make predictions about new, unknown data, resembling the classification problem [66]. The first phase, the training phase, allows classifiers to build the mathematical formula by taking labelled data as input and discriminate among the provided categories via different techniques: for example, support vector machines (SVMs) fit a hyperplane, with the additional aid of kernel functions, to maximise the distance between the closest data observations belonging to different classes [67]; random forests (RF) build a decision tree in which thresholds of feature values determine whether the observation belongs to a class or to another [68]. The following phase is the validation phase, in which the performances of the classifiers are evaluated by the predictions made in a partition of the same training set (cross validation) or in an externally labelled dataset (external validation). The discrepancy between the predicted class and the actual class yields the performance parameters of the model, such as sensitivity (TPR), specificity (TNR), positive predictive value (PPV) and negative predictive value (NPV). Finally, the classifier can be employed for making predictions regarding new data, which can also be weighed according to the performance parameters evaluated in the previous phases. In MSI, an on-tissue classification can be obtained, by generating a MS segmentation image resembling the classification by colouring pixels according to the predicted class.

## 3. Applications in Gastric Cancer

Gastric cancer is a complex, heterogeneous and aggressive disease that represents the third leading cause of cancer-related deaths worldwide [1]. Unfortunately, patients are frequently diagnosed at advanced stages, when the survival outcome is poor [69]. Thus, the discovery of novel drug targets and treatment strategies for patients with advanced GC is the most challenging task in clinical practice.

Recent genomic studies have discovered mutations in the GTPase, Ras homolog family member A (RHOA), that are associated with a poor clinical prognosis in patients with diffuse-type gastric cancers [70,71]. The RHOA signalling pathway activates RHO-associated protein kinases 1 and 2 (ROCK 1/2), which regulate cell contractility, and thus migration and growth may play a role in cancer development [72]. Hisenkamp et al. recently demonstrated that MALDI-MSI could be used to determine the distribution of the drug fasudil to the tumour and surrounding tissues, and to evaluate the pharmacological inhibition of ROCK 1/2 and its effectiveness against gastric cancer in mice [73]. In particular, this study revealed that the parent drug, fasudil, distributed into the stomach and was converted to its active metabolite, hydroxyfasudil. Furthermore, the distribution of the drug in tumorous and non-tumorous tissue was not homogeneous. Fasudil signal intensities were higher in columnar epithelia of the gastric corpus and in parts in the squamous epithelia of the forestomach [73]. There was no obvious enrichment of fasudil or hydroxyfasudil in the tumour areas compared with the non-malignant regions of the stomach. Nevertheless, a significant amount of the drug and its metabolite was able to distribute to the gastrointestinal tumour, suggesting that the drug reached the target organ of interest without being selective for tumour cells [73]. In this analysis, several ion signals were elevated in the tumour region compared to stromal tissue. One signal in particular was then identified as potassium-adducted phophatidylcholine [PC(34:1) + K]^+^ (*m*/*z* 798.541) using FTICR-MS/MS [73]. Similarly, MALDI-MSI has been used to simultaneously map differences in the lipid distribution between gastric cancer lesions and non-neoplastic mucosa whilst preserving the morphological integrity of the analysed tissue. Interestingly, the lipid ion at *m*/*z* 798.5 has been detected in another MALDI-imaging study by Uehara et al., revealing that it was overexpressed in cancer tissue compared to the adjacent non neoplastic mucosa [74]. On the contrary, the intensity of the lipid signal at *m*/*z* 496.3, identified as the proton-adducted lysophosphatidylcholine (LPC) (16:0), was low in cancer lesions [74].

A recent study based on the integrated strategy of MALDI-MSI and immunohistochemical assays investigated the association of cancer progression and the effects of de novo lipogenesis [75]. In fact, it has been reported that high lipogenic activity is one hallmark of tumour cells [76]. In particular, MALDI-FTICR-MSI has been used to analyse lipid localisation in six types of cancer tissue, including 19 samples of gastric cancer (adenocarcinoma), and the results highlighted that the levels of lipids with monounsaturated acyl chains were increased in the cancer microenvironment compared with the adjacent normal tissue, whereas polyunsaturated lipids were decreased in the cancerous area [75].

In addition, MALDI-MSI in negative ion mode has the ability to visualise small molecule metabolites (typically < 500 Da), which are important cellular components closely linked with tumour development and progression [77]. Guo et al. developed an electric field matrix-assisted scanning spraying matrix coating system to deposit matrix on tissue with crystal sizes of <10 μm [78]. The method enabled the in situ detection of cancer-related small molecule metabolites and to visualise their distribution by MALDI-FTICR mass spectrometry imaging on snap-frozen tissues from five gastric cancer patients. It was found that the lipids octadecenoic acid and lysophosphatidylethanolamine (18:1), as well as phosphorylated nucleosides, were significantly upregulated in cancerous areas compared with the adjacent noncancerous areas [78]. On the other hand, nucleosides and *N*-acetylneuraminic acid were significantly decreased in the cancerous area [78].

The poor prognosis of gastric cancer is due to a lack of reliable tumour markers that may improve early-stage diagnosis of cancer. Recently, proteomic approaches using MALDI-MSI techniques have been adopted in order to better understand the pathology and to search for novel diagnostic and therapeutic targets through the characterisation of the proteome profile of a malignant lesion with respect to the non-tumour area.

Deininger et al. employed hierarchical cluster analysis along with principal component analysis in order to uncover proteomic differences in gastric cancer tissue and non-neoplastic stomach mucosa [79]. They demonstrated that histological differences could also be detected on the sole basis of different protein and peptide profiles, thus confirming the reliability of the approach. Furthermore, it was also proposed that MALDI-MSI may be capable of highlighting phenotypic tumour heterogeneity, which cannot be uncovered by using traditional histology [79].

A recent MALDI-MSI study detected seven tumour-specific proteins that predicted unfavourable disease outcome in a cohort of 63 patients with intestinal gastric cancer [80]. Three proteins were identified and successfully validated by immunohistochemistry on an independent set of 118 samples. A protein previously unknown to be implicated in gastric cancer, cysteine-rich intestinal protein 1 (CRIP1), which plays a key role in tumour behaviour, was confirmed to be an independent prognostic factor for gastric cancer. Furthermore, human neutrophil peptide-1 (HNP-1) and S100 calcium binding protein A6 (S100A6) were found to be able to further classify patients with gastric cancer disease at stage I from patients at more advanced stages [80]. Human neutrophil peptides (HNPs) are expressed in neutrophil granules of the innate immune system and are found to be highly expressed in a variety of cancers [81,82,83]. Interestingly, the protein detected at *m*/*z* 3445 (HNP-1) was overexpressed in cancer tissue, confirming the observation of two other MALDI-MSI studies on gastric cancer [84,85], and another that performed MALDI-MS profiling [86]. Besides HNP-1, Cheng et al. demonstrated that HNPs α-defensin 2 and 3 were also overexpressed in gastric cancer tissues and the distribution of HNPs 1–3 overlapped in cancerous tissues, with high abundance in the lamina propria [85]. Similarly, the calcium-binding protein S100A6 was highly expressed in gastric cancer lesions and has also been identified by MALDI-MSI as a potential marker for tumour development of Barrett’s adenocarcinoma [87], known to develop more rapidly than any other gastrointestinal malignancy. Another protein signal, at *m*/*z* 4156, has been observed in the cancer area of intestinal-type gastric cancer and oesophageal adenocarcinoma [84,87], and its role in carcinogenesis and drug resistance was highlighted [88]. Moreover, MALDI-MSI of fresh-frozen Barrett’s adenocarcinoma samples revealed the prognostic role of cytochrome c oxidase subunit 7A2 (COX7A2) and transgelin-2 (TAGLN2) concerning the disease-free survival, whereas the expression of the protein ion at *m*/*z* 11,185, identified as S100 calcium binding protein A10 (S100A10), was an independent prognostic factor [87]. Morita et al. introduced an easy-to-use method for the detection of histological type-specific proteins using a MALDI-MSI approach and 12 FFPE tissue microarrays (TMA) from well, moderately, and poorly differentiated gastric carcinoma samples [89]. Among the detected signals, 54 were classified as signals specific to cancer, with statistically significant differences between adenocarcinoma and normal tissues being observed. The tryptic peptide at *m*/*z* 1325.6 was specifically increased in the poorly differentiated cancer tissue and was identified as histone H4 [89]. Recently, Munteanu et al. employed MALDI-MSI for the in situ analysis of a histone deacetylase inhibitor, the hydroxamic acid panobinostat (LBH-589), focusing on its pharmacodynamic effects in order to visualise the spatiotemporal distribution of acetylated histones and the tumour-selective pharmacodynamic responses in a mouse model of gastrointestinal cancer [90]. Following LBH-589 administration, the nonacetylated (0 Ac) histone H4 was decreased, whereas the acetylated (Ac) H4 states (2 to 4 Ac) were markedly increased in the tumour regions [90].

The MALDI imaging approach in combination with two classification models (support vector machine and random forest) has been promisingly used for gastric cancer tumour classification as well as for the classification of the human epidermal growth factor receptor 2 (HER2/neu) status prediction in gastric cancer [91,92]. Meding et al. were able to classify both the training set and the test set of gastric cancer and Barrett’s adenocarcinoma primary tumour entities with high accuracy [91]. The training set could be classified nearly perfectly, whereas the classification of the test set yielded an accuracy above 94% for Barrett’s cancer and above 88% for gastric cancer with both classifiers [91]. Balluff et al. demonstrated that the HER2/neu status of gastric cancer could be predicted by specific protein patterns originating from breast cancer, with accuracies above 90% independent of the prediction method [92].

Thus far, intratumor heterogeneity is an unresolved factor that influences the evolution of cancer and adversely affects patient outcome. Phenotypically distinct gastric cancer subpopulations have been investigated by MALDI imaging in combination with advanced clustering methods and t-SNE [65,84]. In particular, extensive heterogeneity was noted within and between individual tumour samples. Both studies highlighted two proteins, at *m*/*z* 3445 (HNP-1) and *m*/*z* 14021 (histone H2A), respectively, which were found to be involved in the prognostic signature of the subpopulations [65,84].

Among the post-translational modifications, glycosylation is the most abundant and complex, and alterations in the glycosylation of gastric cancer cells have an impact on gastric carcinogenesis and cancer progression [93,94]. Kunzke et al. investigated in situ native-glycans (*N*-glycans) in 106 primary resected FFPE human gastric cancer tissues by MALDI-MSI in order to understand the underlying molecular mechanisms and discover the clinical implications of glycosylation in gastric cancer [95]. The study pointed out the presence of a glycosaminoglycan fragment (HexNAc-HexA-HexNAc) in tumour stroma regions, an independent prognostic factor for gastric cancer patients [95].

## 4. Concluding Remarks

In the context of gastric cancer, the application of MALDI-MSI is still in its relative infancy. However, there is already sufficient evidence in the literature to suggest that MALDI-MSI can play a crucial role in uncovering the molecular pathways implicated in the development of this particularly deadly cancer.

This approach has already detected molecular alterations associated with gastric cancer at the proteomic, lipidomic, and metabolomic level, and can also monitor the distribution and xenoboiotic metabolism of prospective therapeutic agents such as fasudil. On the basis of these findings, the potential of MALDI-MSI is evident and, by combining these findings using integrative omics approaches, we can improve our understanding of gastric cancer at numerous molecular levels and assist in the clinical management of patients. Whilst this final goal is not imminent, and cannot be achieved using a single approach, MALDI-MSI can certainly make a significant contribution to this pursuit. Nevertheless, the potential of MALDI-MSI for obtaining findings able to contribute to the diagnosis, prognosis, and understanding of numerous diseases, most specifically cancer, is expected to grow in the future as this technology advances.

## Abbreviations

FF	Fresh-frozen
FFPE	Formalin-fixed paraffin-embedded
FTICR	Fourier transform ion cyclotron resonance
GC	Gastric cancer
HCA	Hierarchical clustering analysis
HER2/neu	Human epidermal growth factor receptor 2
HNP	Human neutrophil peptide
MALDI	Matrix-assisted laser desorption/ionisation
MSI	Mass spectrometry imaging
PCA	Principal component analysis
ROCK 1/2	RHO-associated protein kinase 1 and 2
ToF	Time of flight
t-SNE	t-Distributed stochastic neighbour embedding
